# *Bacteroides fragilis* Toxin Induces Extracellular Domain Cleavage of HER2 in Intestinal Epithelial Cells

**DOI:** 10.3390/ijms27198497

**Published:** 2026-09-23

**Authors:** Sang-Hyeon Yoo, Ha-Neul Jang, Da-Hye Kang, Ju-Eun Hong, Jiyun Hong, Young Woo Eom, Ki-Jong Rhee

**Affiliations:** 1 Department of Biomedical Laboratory Science, College of Health Sciences, Yonsei University, Wonju 26493, Republic of Korea; yshyyb@yonsei.ac.kr (S.-H.Y.); zzhaneul0@yonsei.ac.kr (H.-N.J.); dahyekang@missouri.edu (D.-H.K.); jehong07@ecodmlab.com (J.-E.H.); hongjy7pc@yonsei.ac.kr (J.H.); 2 Department of Obstetrics, Gynecology & Women’s Health, University of Missouri School of Medicine, Columbia, MO 65211, USA; 3 Medical Device Business Division, ECO DM LAB Co., Ltd., Cheongju 28121, Republic of Korea; 4 Regeneration Medicine Research Center, Yonsei University Wonju College of Medicine, Wonju 26426, Republic of Korea; yweom@yonsei.ac.kr

**Keywords:** Enterotoxigenic *Bacteroides fragilis*, *B. fragilis* toxin, HER2, colitis, colorectal cancer

## Abstract

Enterotoxigenic *Bacteroides fragilis* (ETBF) secretes *Bacteroides fragilis* toxin (BFT), a metalloprotease that disrupts intestinal epithelial integrity and activates pro-inflammatory signaling. Although E-cadherin cleavage is a well-established response to BFT, the involvement of other membrane receptors in BFT-induced signaling remains poorly defined. In this study, we examined whether HER2 undergoes proteolytic processing following BFT exposure and whether HER2 contributes to BFT-induced ERK activation. In HT29/c1 and MCF-7 epithelial cells, BFT induced the loss of full-length 185 kDa HER2 and the generation of a membrane-tethered ~95 kDa fragment. This truncated HER2 underwent γ-secretase-dependent intramembrane processing and subsequent proteasomal degradation. Knockdown of MMP-7 or ADAM10 did not prevent HER2 cleavage, indicating that neither protease is required for this process. In addition, BFT-induced ERK phosphorylation was observed in HER2-expressing cells but was abolished in HER2-deficient HT29/c1 clones, whereas E-cadherin cleavage and IL-8 secretion remained detectable. These findings identify HER2 as a previously unrecognized target of BFT-induced proteolytic remodeling and support a contribution of HER2 to BFT-induced ERK activation.

## 1. Introduction

Enterotoxigenic *Bacteroides fragilis* (ETBF) has been recognized as a representative pathobiont capable of directly perturbing colonic epithelial integrity and contributing to colorectal tumorigenesis [1,2,3,4]. ETBF carries the *bft* gene, which encodes *B. fragilis* toxin (BFT), a zinc-dependent metalloprotease regarded as its principal virulence factor [5,6]. A consistently observed downstream event following BFT exposure is cleavage of the E-cadherin extracellular domain in epithelial cells [7]. Upon BFT stimulation, epithelial monolayers exhibit loss of adherens junction integrity accompanied by the generation of membrane-tethered E-cadherin fragments that subsequently undergo intramembrane proteolysis followed by proteasomal degradation [8,9]. Earlier biochemical studies failed to demonstrate direct cleavage of isolated E-cadherin by BFT, leading to models in which host-derived proteolytic mechanisms contribute to BFT-induced E-cadherin processing [4,8]. Recently, claudin-4 was identified as a cell-surface receptor for BFT, and BFT binding to claudin-4 was shown to promote BFT-mediated cleavage of the E-cadherin extracellular domain [10].

In parallel with junctional disruption, BFT activates mitogen-activated protein kinase (MAPK) pathways, particularly extracellular signal-regulated kinase (ERK)1/2, and ERK1/2 phosphorylation is detectable within minutes of toxin exposure. These observations indicate that epithelial responses to BFT involve regulated proteolytic processing of membrane-associated proteins together with activation of intracellular signaling pathways. ERK activation is a reproducible feature of BFT signaling; however, the proximal mechanisms linking toxin exposure to MAPK activation remain incompletely defined [11,12].

Epidermal growth factor receptor (EGFR)/ErbB family receptor tyrosine kinases constitute a central signaling axis governing epithelial proliferation and survival. Among these receptors, human epidermal growth factor receptor 2 (HER2) occupies a structurally distinctive position. HER2 extracellular domain adopts an activated, open conformation in the absence of soluble ligand, resulting in persistent exposure of its dimerization arm [13,14,15]. HER2 activation promotes downstream signaling cascades, including the RAS-RAF-MEK-ERK pathway, and sustained ERK phosphorylation represents a recognized consequence of HER2 kinase activity [16,17,18].

Truncation of the HER2 extracellular domain eliminates extracellular regulatory control while preserving fully competent intracellular tyrosine kinase activity. Proteolytic cleavage of full-length HER2 generates a ~105 kDa soluble extracellular domain and a ~95 kDa membrane-anchored fragment composed of the transmembrane segment and intracellular kinase domain [19]. These cleavage-derived truncated HER2 species retain the intracellular catalytic domain and may undergo further intramembrane processing [20]. Cleavage-derived HER2 fragments have been investigated predominantly in breast cancer, where HER2 amplification and targeted therapeutics are well established [21]. HER2 amplification and overexpression have also been documented in colorectal cancer, yet the presence and processing of truncated HER2 species in colorectal epithelia remain insufficiently characterized [22,23,24].

Although E-cadherin cleavage and ERK activation are well-established responses to BFT, the involvement of other membrane receptors in BFT-induced signaling remains poorly defined. Whether HER2 is similarly processed following BFT exposure remains unknown, and the subsequent fate of BFT-induced HER2 fragments has not been determined. In the current study, we investigated whether BFT induces HER2 extracellular domain cleavage and subsequent γ-secretase-dependent intramembrane processing and proteasomal degradation. We also examined the contribution of HER2 to BFT-induced ERK activation using HER2-expressing and HER2-deficient cells. Thus, the current study addresses the involvement of other membrane receptors in BFT-induced signaling by examining HER2 proteolytic processing and its contribution to BFT-induced ERK activation.

## 2. Results

### 2.1. Bacteroides fragilis Toxin-Induced Extracellular Domain Cleavage of HER2 in HT29/c1 and MCF-7 Cells

To determine whether BFT induces extracellular domain cleavage of HER2 and to examine its progression over time, HT29/c1 and MCF-7 epithelial cells were treated with brain heart infusion broth (BHIB), recombinant non-enterotoxigenic *Bacteroides fragilis* (rNTBF; rNT), or recombinant ETBF (rETBF; rET) supernatants (1:10 dilution in serum-free media) for indicated time periods (3 and 6 h). HER2 expression was examined in both cell lysates and culture supernatants by Western blot analysis using antibodies specific to either the intracellular domain or the extracellular domain of HER2. In HT29/c1 cells (Figure 1A), rET treatment led to a reduction in full-length HER2 detected by an intracellular domain-specific antibody, accompanied by the appearance of HER2 extracellular domain fragments of ~105 kDa in the supernatants. No changes were observed in BHIB- or rNT-treated groups, and GAPDH remained stable across conditions. In MCF-7 cells (Figure 1B), rET treatment similarly resulted in a reduction in full-length HER2 detected in cell lysates and concurrent detection of two HER2 extracellular domain-immunoreactive bands in supernatants. The molecular identities of these two bands were not determined in the present study. BHIB and rNT treatments did not alter full-length HER2 levels, and GAPDH expression remained unchanged. These results demonstrate that BFT induces extracellular domain cleavage of HER2 in both HT29/c1 and MCF-7 epithelial cells.

### 2.2. γ-Secretase Cleaves HER2 Intracellular Domain Induced by Bacteroides fragilis Toxin

Because BFT induces γ-secretase-dependent cleavage of the 33 kDa membrane-tethered fragment of E-cadherin in HT29/c1 cells, we evaluated whether γ-secretase also participates in BFT-induced processing of the HER2 intracellular domain in HT29/c1 and MCF-7 cells. Cells were pre-treated with a γ-secretase inhibitor (L-685,458) at indicated concentrations (0 nM, 1.6 nM, 8 nM, 20 nM, 0.2 μM, 1 μM, and 5 μM) for 30 min in serum-free media and subsequently incubated with BHIB, rNT, or rET supernatants (1:10 dilution) for 9 h. Western blot analysis using an HER2 intracellular domain-specific antibody showed that rET treatment reduced full-length HER2 (185 kDa) regardless of inhibitor concentration, while a 95 kDa membrane-associated HER2 intracellular domain fragment (p95HER2) was detected only under γ-secretase-inhibited conditions (Figure 2A,B). In HT29/c1 cells, accumulation of p95HER2 was most evident at 1–5 μM of L-685,458. A similar pattern was observed in MCF-7 cells, where p95HER2 was detected at 0.2–5 μM.

Based on these results, 5 μM of L-685,458 was selected for subsequent time-course experiments. The 3 and 9 h time points were selected to compare earlier and later stages of HER2 intracellular processing, with the 9 h time point allowing sufficient accumulation of cleavage-derived intracellular fragments. Cells were pre-treated with 5 μM of L-685,458 for 30 min and then exposed to BHIB, rNT, or rET for 3 or 9 h. Western blot analysis showed that full-length HER2 decreased after 9 h of rET treatment in all groups; however, p95HER2 accumulated only in cells co-treated with L-685,458 and rET. BHIB- and rNT-treated controls retained full-length HER2 without detectable cleavage fragments. These findings demonstrate that γ-secretase participates in BFT-induced intracellular processing of HER2, resulting in accumulation of p95HER2 in both HT29/c1 and MCF-7 epithelial cells.

### 2.3. Proteasome Degrades HER2 Intracellular Domain Induced by Bacteroides fragilis Toxin

To determine whether proteasome participates in degradation of HER2 intracellular domain following BFT exposure, HT29/c1 and MCF-7 epithelial cells were pre-treated with proteasome inhibitor bortezomib at indicated concentrations (0 nM, 1.28 nM, 6.4 nM, 32 nM, 0.16 μM, 0.8 μM, and 4 μM) for 30 min in serum-free media and subsequently incubated with BHIB, rNT, or rET supernatants (1:10 dilution) for 9 h. Western blot analysis using an HER2 intracellular domain-specific antibody showed that rET treatment resulted in accumulation of an 80 kDa HER2 intracellular domain fragment under proteasome-inhibited conditions, accompanied by reduction in full-length HER2 (185 kDa). BHIB- and rNT-treated groups maintained full-length HER2 without a detectable 80 kDa band. In HT29/c1 cells, the 80 kDa fragment appeared from 32 nM bortezomib and remained detectable up to 4 μM. In MCF-7 cells, the 80 kDa band was observed between 0.16 μM and 4 μM, with stronger accumulation at 4 μM (Figure 3A,B).

To further evaluate HER2 intracellular processing at the earlier and later time points, cells were pre-treated with 4 μM of bortezomib for 30 min and then exposed to BHIB, rNT, or rET for 3 or 9 h. Western blot analysis showed that full-length HER2 decreased after 9 h of rET treatment, and an 80 kDa HER2 intracellular domain fragment accumulated in rET and bortezomib co-treated cells. At 3 h, a faint 80 kDa band was detectable, whereas at 9 h the band intensity increased. BHIB- and rNT-treated groups showed no detectable intracellular domain fragments. As a reference control, γ-secretase inhibition by L-685,458 produced a 95 kDa band corresponding to membrane-tethered p95HER2, consistent with previous results. In contrast, proteasome inhibition resulted in accumulation of the smaller 80 kDa fragment (Figure 3A,B). These findings demonstrate that the proteasome mediates degradation of the 80 kDa HER2 intracellular domain fragment generated following BFT-induced processing, indicating sequential γ-secretase-dependent cleavage followed by proteasome-dependent degradation.

### 2.4. Bacteroides fragilis Toxin-Induced HER2 Cleavage Does Not Require MMP-7 or ADAM 10

Both matrix metalloproteinase-7 (MMP-7) and a disintegrin and metalloproteinase 10 (ADAM10) mediate E-cadherin extracellular domain shedding in select epithelial cells [25,26,27,28]. In addition, ADAM10 has been identified as a major source of HER2 extracellular domain sheddase activity in HER2-overexpressing breast cancer cells [29]. To evaluate whether MMP-7 or ADAM10 contributes to BFT-induced HER2 cleavage, HT29/c1 epithelial cells were subjected to siRNA-mediated knockdown of each protease. Cells were transfected with scrambled siRNA or siRNA against MMP-7 or ADAM10 for 24 h, followed by treatment with rNT or rET supernatants (1:10 dilution in serum-free media) for 6 h. The 6 h treatment period was selected to evaluate HER2 cleavage under the same treatment window used for the HER2 extracellular domain cleavage analysis. Western blot analysis confirmed efficient knockdown, as expression of MMP-7 or ADAM10 was markedly reduced in siRNA-transfected samples compared with SFM- and scramble-treated controls (Figure 4A,B). These results verified that endogenous expression of each protease was sufficiently depleted to assess its contribution to HER2 cleavage. Despite knockdown of either protease, rET treatment consistently reduced full-length HER2 in SFM-, scramble-, MMP-7 siRNA-, and ADAM10 siRNA-treated groups (Figure 4A,B). In contrast, all rNT-treated conditions retained full-length HER2 without detectable cleavage fragments. These findings indicate that HER2 cleavage persisted even when MMP-7 or ADAM10 expression was depleted, demonstrating that neither protease is required for BFT-induced HER2 cleavage.

### 2.5. HER2 Knockout Validation and Differential Contribution of HER2 to Bacteroides fragilis Toxin-Induced Cellular Responses

To establish a cellular model for evaluating the contribution of HER2 to BFT-induced responses, HER2 knockout (KO) HT29/c1 cells were generated using CRISPR-Cas9 targeting of the ERBB2 locus. Two independent clones (KO#1 and KO#2) were selected. Western blot analysis confirmed complete loss of full-length HER2 in both KO clones, whereas wild-type (WT) cells displayed a distinct HER2 band (Figure 5A). Immunofluorescence confocal imaging further demonstrated absence of membrane-associated HER2 staining in KO#1 and KO#2 cells compared with WT cells (Figure 5B). To determine whether HER2 deletion elicited compensatory changes in other HER family members, EGFR and HER3 expression was examined. EGFR expression remained unchanged across WT and KO clones, and HER3 showed a similar reduction in response to rET in both WT and HER2-deficient cells (Figure 5C). These results verified successful generation of HER2 KO HT29/c1 cells without compensatory alteration of other HER receptors.

Because E-cadherin cleavage and interleukin-8 (IL-8) secretion are well-established cellular responses to BFT exposure, these responses were examined in WT, KO#1, and KO#2 cells. For E-cadherin analysis, cells were treated with rNT or rET for 6 h. Western blot analysis showed marked reduction in full-length E-cadherin in all rET-treated groups, indicating that E-cadherin cleavage occurred independently of HER2 expression (Figure 5C). For IL-8 analysis, cells were treated with rNT or rET for 24 h to allow secreted IL-8 to accumulate in the culture supernatant for ELISA measurement. rET treatment significantly increased IL-8 secretion compared with rNT treatment in WT (*p* = 0.0035), KO#1 (*p* < 0.0001), and KO#2 cells (*p* = 0.0088). Upon rET treatment, IL-8 levels were significantly lower in KO#1 cells than in WT cells (*p* = 0.0086), whereas no significant difference was observed between KO#2 and WT cells (*p* = 0.8141).

To assess whether HER2 contributes to BFT-induced ERK activation, WT and HER2 KO cells were treated with rNT or rET for 40 min. Total ERK levels remained constant across all groups. In WT cells, rET treatment strongly induced ERK phosphorylation, whereas KO#1 and KO#2 cells failed to exhibit detectable p-ERK under identical conditions (Figure 5E). These findings support a differential contribution of HER2 to BFT-induced cellular responses, linking HER2 expression more closely to ERK activation than to E-cadherin cleavage or IL-8 induction.

## 3. Discussion

This study demonstrates that under BFT exposure, HER2 does not undergo nonspecific damage but instead undergoes defined structural remodeling. In both HT29/c1 and MCF-7 epithelial cells, cleavage of the HER2 extracellular domain occurred first, followed by γ-secretase-mediated intramembrane cleavage and subsequent proteasomal degradation of the resulting intracellular fragment (Figure 1). This sequential processing is consistent with established features of regulated intramembrane proteolysis and indicates that BFT directs HER2 into a coordinated proteolytic pathway rather than causing incidental destabilization [20,30,31,32]. Analysis of downstream responses further revealed distinct patterns of BFT-induced cellular responses in relation to HER2 status. E-cadherin cleavage and IL-8 secretion remained detectable in HER2-deficient cells. Specifically, rET-induced IL-8 secretion was significantly increased compared with rNT treatment in both WT and HER2-deficient cells, indicating that IL-8 induction was maintained following HER2 loss. However, when the magnitude of rET-induced IL-8 secretion was compared between WT and HER2-deficient cells, a significant reduction was observed in KO#1 cells but not in KO#2 cells. Because phenotypic differences observed in a single knockout clone may reflect clone-specific effects, independent knockout clones are important for assessing the reproducibility of the phenotype. Accordingly, we examined two independent HER2 knockout clones, KO#1 and KO#2. Based on the results from both clones, HER2 deficiency did not consistently affect the extent of rET-induced IL-8 secretion compared with WT cells.

In contrast, BFT-induced ERK phosphorylation was observed only in cells retaining HER2 expression. These findings associate HER2 expression with the BFT-induced ERK response but do not establish a direct HER2-to-ERK signaling mechanism.

BFT has previously been shown to induce E-cadherin cleavage, adherens junction disruption, β-catenin translocation, and the production of chemokines such as IL-8 [7,12,33]. These events provide a structural and inflammatory framework for ETBF-mediated epithelial perturbation. The findings of this study do not revise these canonical mechanisms. Rather, under the same stimulation conditions that produce junctional disruption and inflammatory responses, HER2 was also shown to undergo regulated structural processing. Therefore, HER2 modification should be interpreted not as a replacement for established BFT signaling pathways but as an additional receptor-level event that expands the known spectrum of BFT-induced epithelial responses.

The proximal mechanism responsible for BFT-induced HER2 extracellular domain cleavage remains unresolved. Depletion of MMP-7 or ADAM10 did not prevent BFT-induced loss of full-length HER2, indicating that neither protease is required for this process. Notably, ADAM10 has been identified as a major source of HER2 extracellular domain sheddase activity in HER2-overexpressing breast cancer cells [29], whereas BFT-induced loss of full-length HER2 persisted after ADAM10 knockdown in HT29/c1 cells. This difference suggests that BFT-associated HER2 cleavage may not follow the same ADAM10-dependent mechanism described in HER2-overexpressing breast cancer cells. These findings exclude MMP-7 and ADAM10 as essential mediators but do not identify the proteolytic event that initiates HER2 extracellular domain cleavage. In light of recent evidence that claudin-4 enables BFT-mediated E-cadherin cleavage [10], whether HER2 cleavage is caused directly by BFT, depends on claudin-4 or another cell-surface factor, or is mediated through a distinct host-derived proteolytic pathway remains to be determined.

The BFT-induced structural transition of HER2 may also have biological implications for receptor regulation. Loss of the extracellular domain generates a membrane-tethered truncated form, p95HER2, which retains the intracellular kinase domain prior to further processing. In this study, the intracellular fragment generated following γ-secretase cleavage was predominantly short-lived and rapidly eliminated through proteasomal degradation. However, the rapid turnover of this fragment does not exclude the possibility that transient HER2 intermediates have functional consequences. As reported in breast cancer models, HER2 can generate a membrane-tethered p95HER2 form through loss of the extracellular domain while retaining the intracellular kinase domain, and this truncated receptor has been associated with altered signaling properties and reduced responsiveness to HER2-targeted therapies. Accordingly, transient accumulation of p95HER2 may create a structurally distinct receptor state before subsequent intramembrane cleavage and degradation, potentially influencing downstream signaling during this interval [19,34,35,36]. Thus, whereas previous studies have focused largely on the biological properties of truncated HER2 species, our findings further define the downstream fate of the BFT-induced ~95 kDa fragment as part of a sequential processing pathway that proceeds through additional intramembrane processing and degradation. Loss of the extracellular domain may therefore transiently remove extracellular regulatory constraints while preserving the kinase domain until further processing occurs [13,37]. Under repeated or sustained BFT exposure, extracellular cleavage and intramembrane processing may recurrently generate such cleaved receptor states. These repeated structural transitions could transiently alter the availability of HER2-dependent signaling without requiring HER2 gene amplification or overexpression. From this perspective, HER2 cleavage under BFT stimulation represents regulated structural remodeling rather than simple receptor destabilization.

ERK phosphorylation was evaluated at a single time point rather than across an extended temporal series. Because ERK activation is a reproducible response to BFT exposure while its proximal regulation remains incompletely defined [11,12], this approach was used to compare the ERK response between HER2-expressing and HER2-deficient cells under matched BFT stimulation conditions, rather than to define complete kinetics of MAPK signaling. Prior reports demonstrating that ERK phosphorylation reaches a peak approximately 40 min after BFT exposure provided the rationale for selecting this time point [12]. The loss of BFT-induced ERK phosphorylation in HER2-deficient clones supports a contribution of HER2 to this response. However, because phosphorylated HER2 was not directly evaluated and ERK phosphorylation was examined at a single time point, the present data do not establish whether HER2 directly mediates BFT-induced ERK activation or whether HER2 deletion indirectly alters the ERK response.

The selection of cellular models also warrants consideration. HT29/c1 cells were selected as an intestinal epithelial model because HT29-derived cells are among the most established models for studying BFT and are highly responsive to BFT [10]. MCF-7 cells are generally classified as HER2-negative breast cancer cells; however, they retain detectable endogenous HER2 expression. HER2 protein has been detected by Western blot analysis in control MCF-7 cells [18]. In addition, MCF-7 cells have been characterized as estrogen receptor-positive and HER2-low, and HER2 expression in these cells has been evaluated at both the mRNA and protein levels by real-time RT-PCR and Western blot analysis [38]. HER2 expression in MCF-7 cells is also subject to regulation, as ERβ1 overexpression has been reported to increase HER2 expression at both the mRNA and protein levels [39]. Moreover, BFT-induced E-cadherin cleavage and subsequent proteasomal degradation have previously been demonstrated in MCF-7 cells [9], supporting the responsiveness of this cell line to BFT. More recently, ETBF and secreted BFT-1 have also been reported to promote breast cancer cell stemness and chemoresistance [40]. Inclusion of MCF-7 alongside HT29/c1 therefore enabled evaluation of whether BFT-induced HER2 processing was confined to the intestinal epithelial context or could also occur in a distinct epithelial cell type. Observation of extracellular truncation and sequential processing in both cell lines supports the interpretation that HER2 remodeling is not restricted to a single epithelial model.

Finally, several caveats regarding HER2 signaling should be considered. Phosphorylated HER2 was not directly evaluated, and the intracellular localization of cleavage-derived fragments was not examined. Although BFT-induced ERK phosphorylation was abolished in both HER2-deficient clones, our results do not determine whether the loss of ERK phosphorylation reflects a direct contribution of HER2 to BFT-induced ERK signaling or an indirect effect associated with HER2 deletion. Therefore, the relationship between HER2 and BFT-induced ERK activation remains to be further defined. In addition, these findings reflect molecular events observed in epithelial cell lines and therefore require validation in tissue-based models. Nevertheless, the controlled experimental system employed here enabled focused delineation of BFT-induced HER2 structural transitions and their association with intracellular signaling responses.

## 4. Materials and Methods

### 4.1. Cell Culture

Human intestinal epithelial cells (HT29/c1) and human breast epithelial cells (MCF-7) were utilized in this study. Both cell lines were maintained in Dulbecco’s modified Eagle’s medium (DMEM; Corning, Manassas, VA, USA) supplemented with 5% fetal bovine serum (Gibco, Grand Island, NY, USA), 10 mM HEPES (Gibco, Inchinnan, Scotland, UK), 100 U/mL penicillin (Gibco, Grand Island, NY, USA), and 100 μg/mL streptomycin (Gibco, Grand Island, NY, USA). Cells were cultured at 37 °C in humidified incubator with 5% CO_2_.

### 4.2. Bacterial Strains and Culture Conditions

Recombinant *Bacteroides fragilis* strains derived from a nontoxigenic wild-type background (NTBF) were used. Strains engineered to express active bft-2 (rETBF) or a catalytically inactive mutant (bft-2 E349A; rNTBF) were kindly provided by Cynthia Sears and Augusto Franco (Johns Hopkins University, Baltimore, MA, USA). Both recombinant strains harbor plasmid pFD340 encoding clindamycin resistance as a selection marker. Bacterial cultures were maintained on brain heart infusion (BHI) (MB cell, Seoul, Republic of Korea) agar and in BHI broth supplemented with hemin (5 μg/mL; Sigma-Aldrich, St. Louis, MO, USA), L-cysteine (0.5 mg/mL; Sigma-Aldrich, St. Louis, MO, USA), clindamycin (6 μg/mL; Tokyo Chemical Industry, Tokyo, Japan), and gentamicin (50 μg/mL; Corning, Manassas, NY, USA). Cultures were incubated anaerobically at 37 °C for 48 h using GasPak EZ anaerobe container system sachets (BD Biosciences, Franklin Lakes, NJ, USA).

### 4.3. Collection of Bacterial Supernatants

Recombinant *Bacteroides fragilis* strains (rETBF and rNTBF) were streaked on supplemented BHI agar and incubated anaerobically at 37 °C for 48 h. A single colony was inoculated into supplemented BHI broth and cultured for 24 h under identical anaerobic conditions. Cultures were centrifuged at 14,000 *g* for 10 min at 4 °C. Supernatants were collected and filtered through 0.45 μm syringe filters (Sartorius, Goettingen, Germany). Filtered supernatants were stored at −80 °C until use. For stimulation experiments, supernatants were diluted 1:10 in serum-free medium (SFM).

### 4.4. Inhibitor Study

For inhibition of γ-secretase and proteasome activity, cells were pre-treated with the γ-secretase inhibitor L-685,458 (APExBIO, Houston, TX, USA) or the proteasome inhibitor bortezomib (Selleck Chemical LLC, Houston, TX, USA). Stock solutions were prepared in DMSO and diluted in SFM to a final concentration of 5 μM immediately before use. Cells were pre-incubated with inhibitors for 30 min at 37 °C prior to stimulation. BHIB control supernatant, rNTBF supernatant, or rETBF supernatant diluted 1:10 in SFM were applied in the presence or absence of inhibitors. Following treatment, cells were lysed for Western blot analysis.

### 4.5. Western Blot Analysis

Cells were washed with Dulbecco’s phosphate-buffered saline (DPBS; Welgene, Seoul, Republic of Korea) and lysed in radioimmunoprecipitation assay (RIPA) buffer (Sigma-Aldrich, St. Louis, MO, USA) containing protease and phosphatase inhibitors (Roche Diagnostics, Mannheim, Germany). Protein concentrations were measured using Pierce™ BCA Protein Assay Kit (Thermo Scientific, Rockford, IL, USA). Equal amounts of protein were separated by SDS-polyacrylamide gel electrophoresis (SDS-PAGE) and transferred to nitrocellulose membranes (Pall, Port Washington, NY, USA, or Bio-Rad, Hercules, CA, USA). Membranes were blocked with 5% skim milk in TBS-T and incubated with primary antibodies overnight at 4 °C, followed by horseradish peroxidase (HRP)-conjugated secondary antibodies (Jackson ImmunoResearch, West Grove, PA, USA). The primary antibodies used in this study included anti-HER2 (extracellular domain; MS-1350-P1, Thermo Scientific, Rockford, IL, USA), anti-HER2 (intracellular domain; 2165, Cell Signaling Technology, Danvers, MA, USA), anti-MMP-7 (sc-80205, Santa Cruz Biotechnology, Dallas, TX, USA), anti-ADAM10 (2051, ProSci, Poway, CA, USA), anti-E-cadherin (intracellular domain; 610108, BD Biosciences, Franklin Lakes, NJ, USA), anti-EGFR (4267, Cell Signaling Technology, Danvers, MA, USA), anti-HER3 (12708, Cell Signaling Technology, Danvers, MA, USA), anti-ERK1/2 (4695, Cell Signaling Technology, Danvers, MA, USA), anti-phospho-ERK1/2 (4370, Cell Signaling Technology, Danvers, MA, USA), and anti-GAPDH (CB1001, Calbiochem, San Diego, CA, USA). Immunoreactive bands were detected using enhanced chemiluminescence (ECL) detection reagents (Bio-Rad, Hercules, CA, USA or Thermo Scientific™, Rockford, IL, USA).

### 4.6. Small-Interference RNA (siRNA) Transfection

HT29/c1 cells were seeded in 24-well plates at a density of 2 × 10^5^ cells per well in antibiotic-free and serum-free DMEM and incubated at 37 °C. siRNA targeting MMP7 or ADAM10 (Dharmacon, Lafayette, CO, USA) was transfected using DharmaFECT 4 reagent (Dharmacon, Lafayette, CO, USA) according to the manufacturer’s instructions. After overnight incubation with siRNA-transfection complexes, the culture medium was replaced with DMEM containing 10% fetal bovine serum (Gibco, Grand Island, NY, USA), and cells were maintained for the indicated time to allow gene knockdown. Cells were subsequently treated with rETBF or rNTBF supernatants diluted 1:10 in serum-free medium and processed for Western blot analysis.

### 4.7. CRISPR-Cas9 Gene Knockout

To generate ERBB2 (HER2) knockout HT29/c1 cell lines, two single guide RNAs (sgRNAs) targeting exon 8 of ERBB2 were designed using CRISPOR (crispor.tefor.net). sgRNA sequences were as follows: ERBB2 KO #1 forward 5′-CAC CGT GAT AGA CAC CAA CCG CTC T-3′, reverse 5′-AAA CAG AGC GGT TGG TGT CTA TCA C-3′; ERBB2 KO #2 forward 5′-CAC CGA TAG ACA CCA ACC GCT CTC-3′, reverse 5′-AAA CGA GAG CGG TTG GTG TCT ATC-3′. Oligonucleotides were synthesized by Cosmogenetech (Seoul, Republic of Korea). Annealed sgRNAs were ligated into Esp3I (BsmBI)-digested lentiCRISPR v2 vector (Thermo Fisher Scientific, Waltham, MA, USA). For lentiviral production, sgRNA-lentiCRISPR v2 plasmids were co-transfected with pMD2.G and psPAX2 packaging plasmids (Addgene, Cambridge, MA, USA) into HEK293T cells using Lipofectamine 2000 (Invitrogen, Carlsbad, CA, USA). Viral supernatants were collected, filtered, and used to infect HT29/c1 cells in the presence of polybrene (0.08 μg/mL). Following infection, cells were selected with puromycin (1 μg/mL; Sigma-Aldrich, St. Louis, MO, USA) to establish stable ERBB2 knockout populations. Lentiviral experiments were conducted under approval of the Institutional Biosafety Committee of Yonsei University MIRAE Campus (IBC No. 201908-P-004-01).

### 4.8. Immunofluorescence Staining and Confocal Microscopy

Cells were cultured on sterilized glass coverslips and fixed with 4% paraformaldehyde (Biosesang, Seongnam, Republic of Korea) for 20 min at room temperature. Following fixation, cells were washed with Dulbecco’s phosphate-buffered saline (DPBS; Welgene, Seoul, Republic of Korea) and permeabilized with cold absolute methanol (Merck, Darmstadt, Germany) at −20 °C for 10 min. Cells were blocked in 5% normal goat serum in 0.2% Triton X-100/DPBS for 1 h at room temperature and incubated with primary antibodies diluted in 0.1% Triton X-100/DPBS overnight at 4 °C. Alexa Fluor 568-conjugated secondary antibody (1:1000; Invitrogen, Carlsbad, CA, USA) was applied for 1 h at room temperature. After washing with DPBS, coverslips were mounted using mounting medium containing DAPI (VECTOR Laboratories, Peterborough, UK). Fluorescence images were acquired using a Carl Zeiss LSM710 confocal microscope (Carl Zeiss, Oberkochen, Germany) and processed using ZEN 2.5 software (Carl Zeiss, Oberkochen, Germany) and ImageJ software (version 1.53, NIH, Bethesda, MD, USA)

### 4.9. Cytokine Analysis

Cell culture supernatants were collected and stored at −80 °C. IL-8 concentrations were measured using a human IL-8 ELISA kit (R&D Systems, Minneapolis, MN, USA) according to the manufacturer’s protocol.

### 4.10. Statistical Analysis

Data are presented as mean ± standard error of the mean (SEM). Statistical analyses were performed using GraphPad Prism 9.3.1 (GraphPad Software, San Diego, CA, USA). Comparisons between groups were conducted using unpaired t-tests. *P*-values < 0.05 were considered statistically significant.

## 5. Conclusions

This study defines a sequential processing pathway in which HER2 undergoes extracellular truncation, γ-secretase-mediated intramembrane cleavage, and proteasomal degradation of intracellular fragments following BFT stimulation. These structural transitions indicate that HER2 is directed into a regulated processing cascade rather than subjected to nonspecific degradation. Comparison of HER2-expressing and HER2-deficient cells showed that E-cadherin cleavage and IL-8 secretion remained detectable. Collectively, these findings establish that BFT induces coordinated structural remodeling of HER2 and support the contribution of HER2 to BFT-induced ERK activation. However, the relationship between HER2 and ERK activation remains to be further defined. This work provides a mechanistic framework for understanding HER2 structural modification under toxin-induced epithelial stress. Further studies using additional epithelial cell lines and tissue-based models will be required to determine whether these findings extend beyond the cellular models examined here.

## Figures and Tables

**Figure 1:**
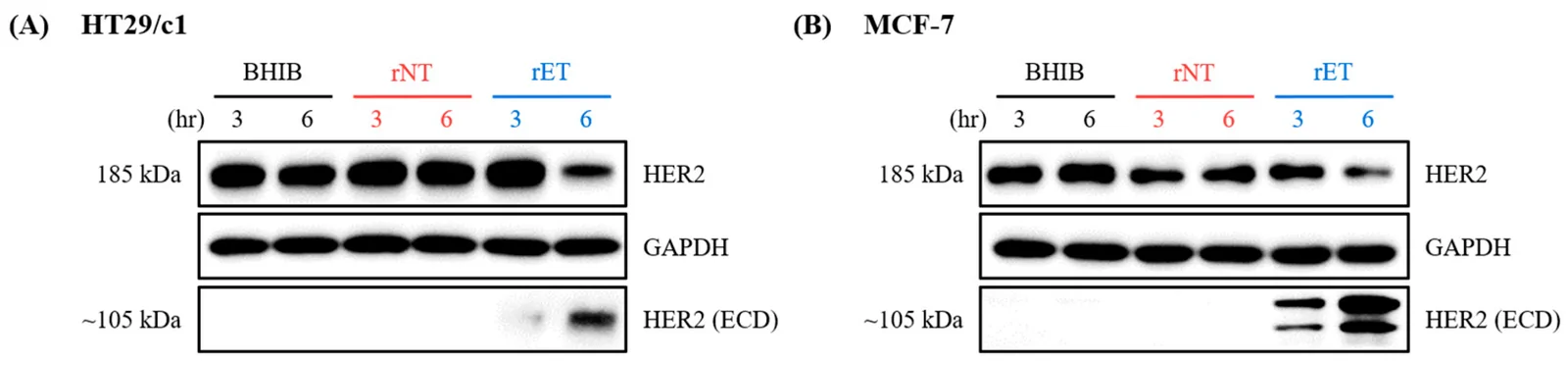
BFT induces HER2 extracellular domain cleavage in HT29/c1 and MCF-7 cells. HT29/c1 (**A**) and MCF-7 (**B**) cells were incubated with BHIB, rNT, or rET supernatants (1:10 dilution in serum-free media) for 3 and 6 h. HER2 expression was examined in both cell lysates and culture supernatants by Western blot analysis using antibodies specific to the intracellular domain or extracellular domain of HER2. Reduction in full-length HER2 in cell lysates and detection of ~105 kDa extracellular domain fragments in supernatants were observed following rET treatment. BHIB and rNT groups served as controls. GAPDH was used as a loading control.

**Figure 2:**
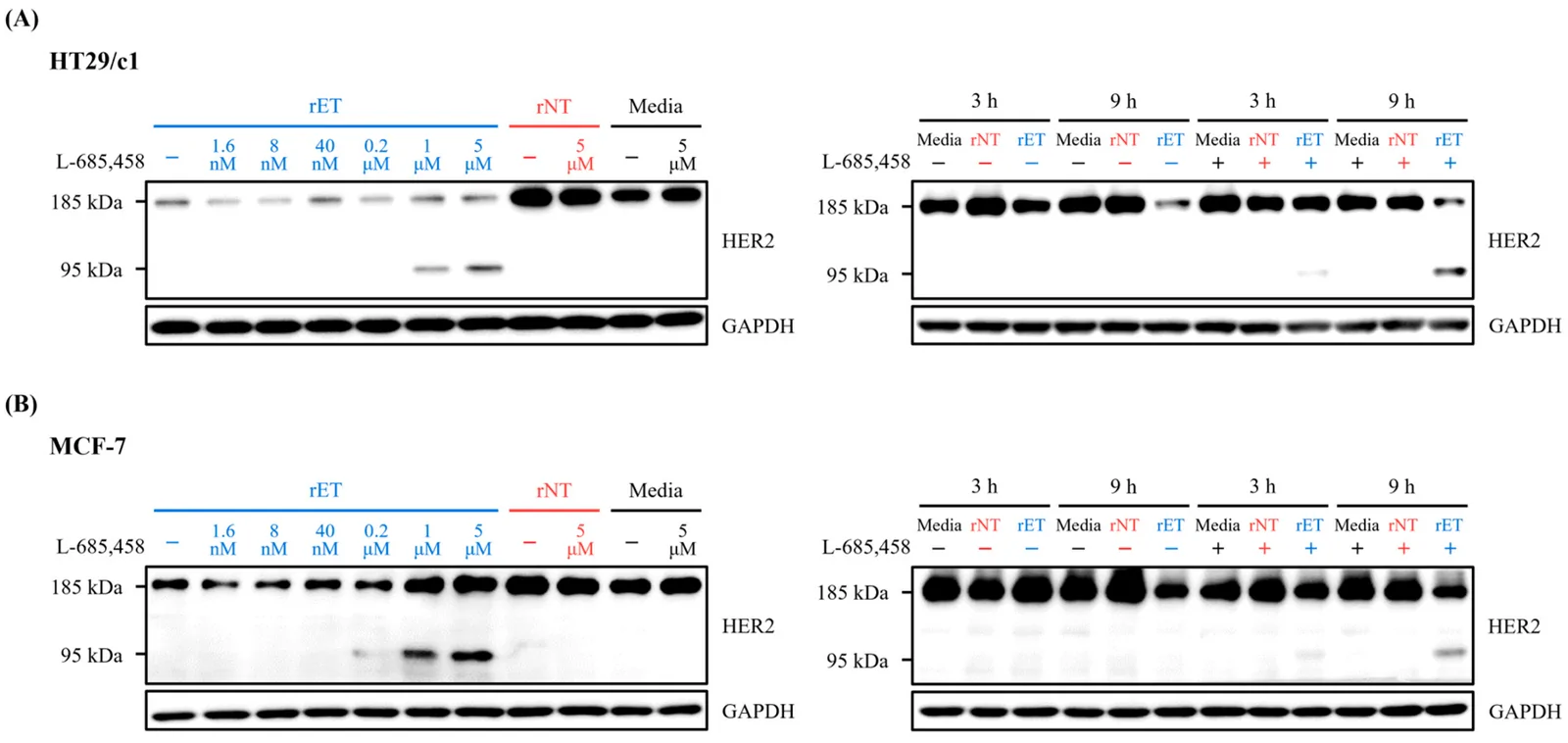
γ-Secretase inhibition leads to accumulation of membrane-tethered HER2 intracellular domain following BFT treatment in HT29/c1 and MCF-7 cells. HT29/c1 (**A**) and MCF-7 (**B**) cells were pre-treated with the indicated concentrations of γ-secretase inhibitor L-685,458 (0, 1.6 nM, 8 nM, 20 nM, 0.2 μM, 1 μM, and 5 μM) for 30 min in serum-free media and subsequently incubated with BHIB, rNT, or rET supernatants (1:10 dilution) for 9 h. In time-course experiments, cells were pre-treated with 5 μM of L-685,458 and incubated with BHIB, rNT, or rET for 3 or 9 h. Cell lysates were analyzed by Western blot using an HER2 intracellular domain-specific antibody. Accumulation of 95 kDa HER2 intracellular domain fragment (p95HER2) was observed under γ-secretase-inhibited conditions following rET treatment. BHIB- and rNT-treated groups served as controls. GAPDH was used as a loading control.

**Figure 3:**
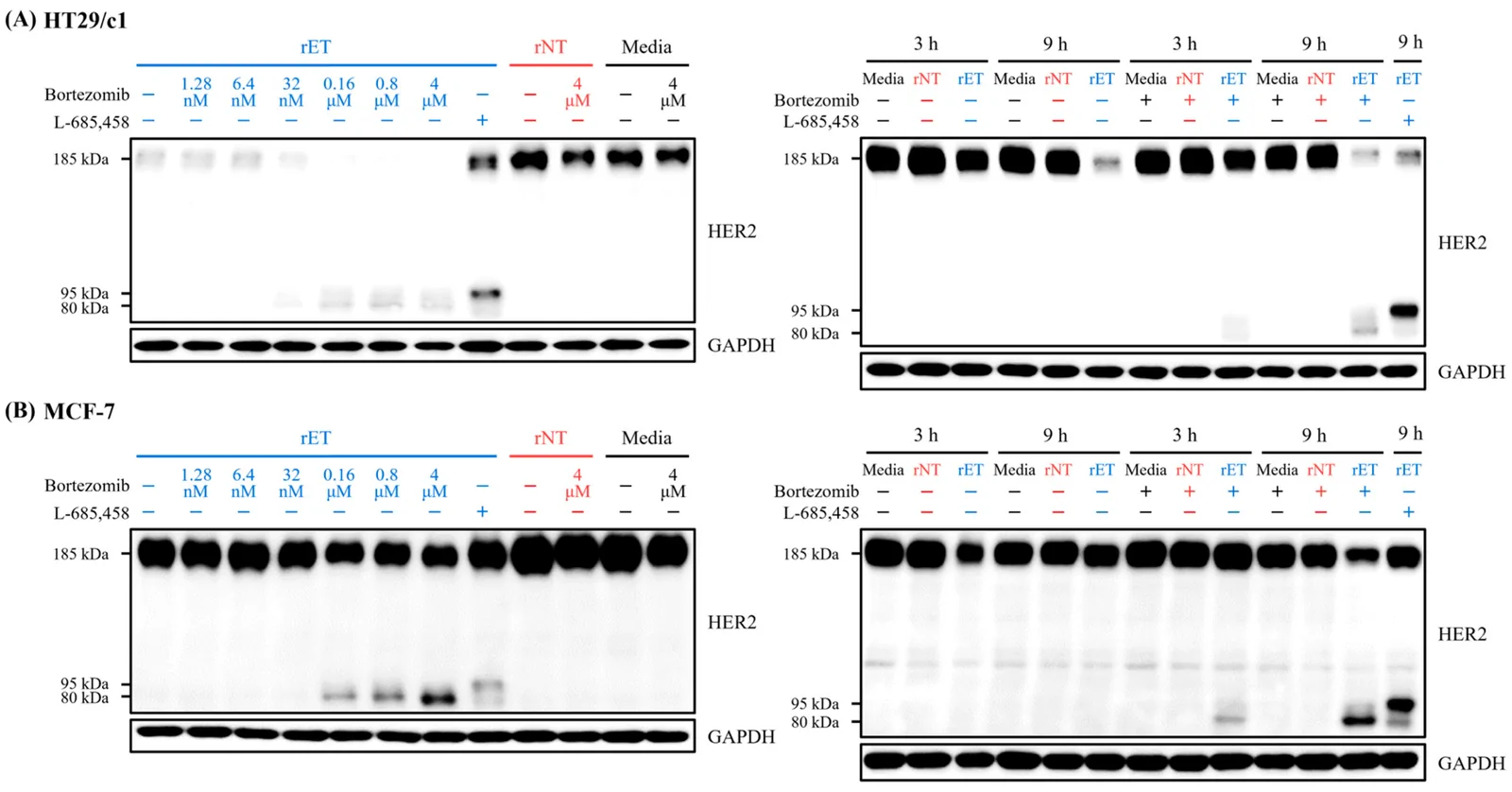
Proteasome inhibition leads to accumulation of the 80 kDa HER2 intracellular domain fragment following BFT treatment in HT29/c1 and MCF-7 cells. HT29/c1 (**A**) and MCF-7 (**B**) cells were pre-treated with the indicated concentrations of the proteasome inhibitor bortezomib (0, 1.28 nM, 6.4 nM, 32 nM, 0.16 μM, 0.8 μM, and 4 μM) for 30 min in serum-free media and subsequently incubated with BHIB, rNT, or rET supernatants (1:10 dilution) for 9 h. In time-course experiments, cells were pre-treated with 4 μM of bortezomib and incubated with BHIB, rNT, or rET for 3 or 9 h. Cell lysates were analyzed by Western blot using an HER2 intracellular domain-specific antibody. Accumulation of the 80 kDa HER2 intracellular domain fragment was observed under proteasome-inhibited conditions following rET treatment. γ-Secretase inhibition produced a 95 kDa p95HER2 band as a reference control. BHIB- and rNT-treated groups served as controls. GAPDH was used as a loading control.

**Figure 4:**
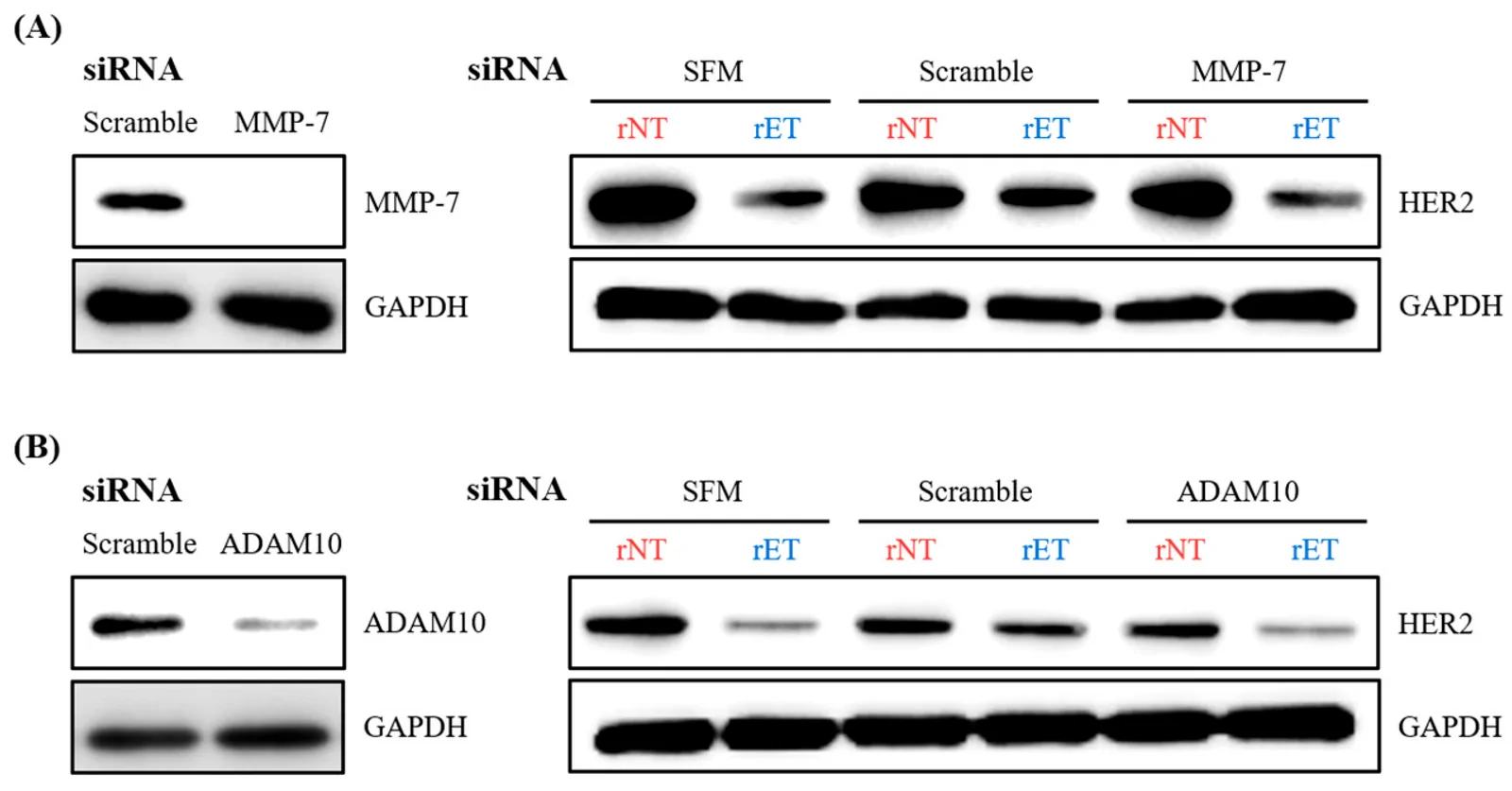
BFT-induced HER2 cleavage is not prevented by MMP-7 or ADAM10 knockdown in HT29/c1 cells. HT29/c1 cells were transfected for 24 h with scrambled siRNA, MMP-7 siRNA (**A**), or ADAM10 siRNA (**B**), followed by incubation with rNT or rET supernatants (1:10 dilution in serum-free media) for 6 h. Cell lysates were analyzed by Western blot for MMP-7 or ADAM10 to confirm knockdown efficiency and for HER2 to assess cleavage. Reduction in full-length HER2 was observed in rET-treated cells regardless of protease depletion. rNT-treated groups served as controls. GAPDH was used as a loading control.

**Figure 5:**
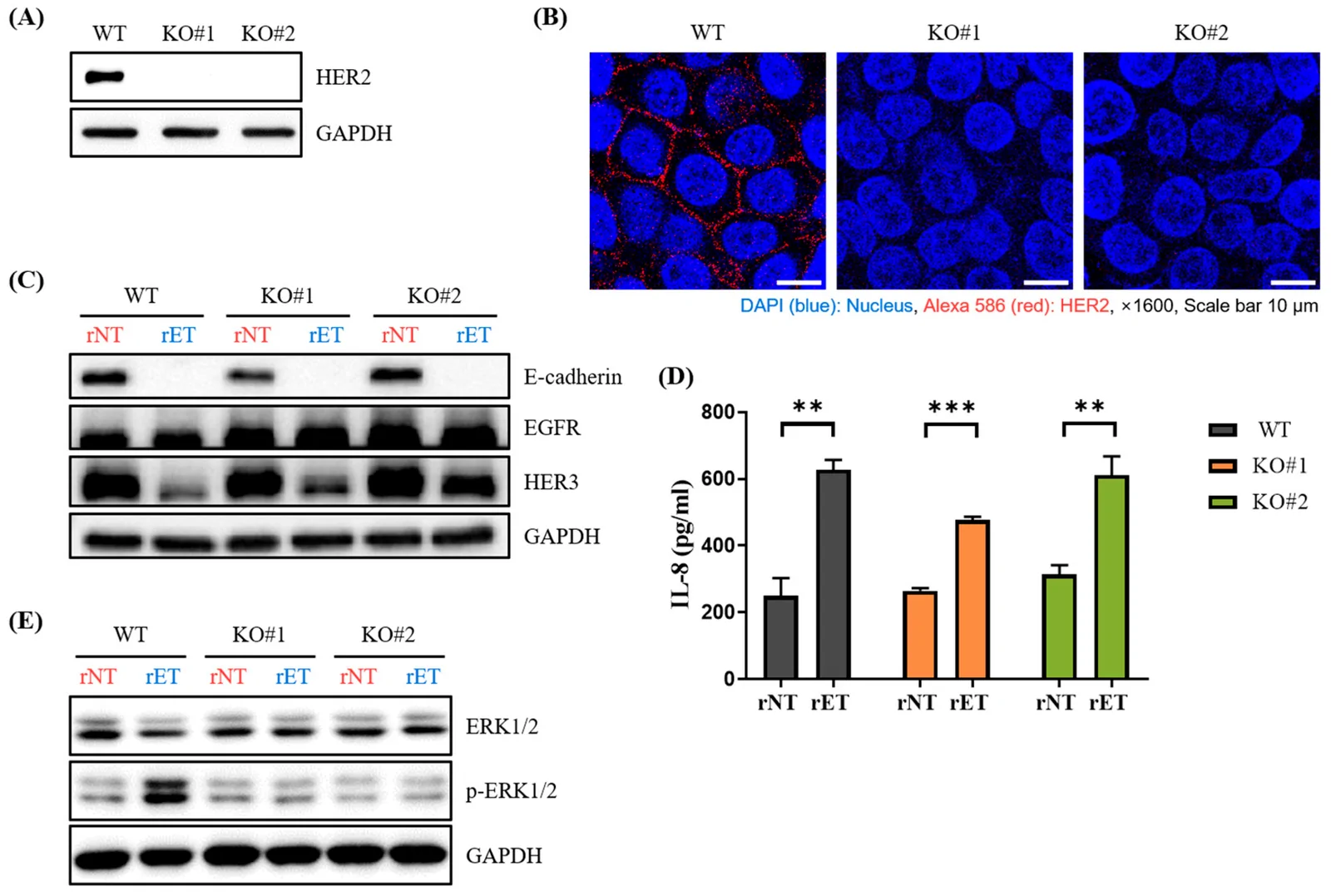
Validation of HER2 knockout and differential contribution of HER2 to BFT-induced cellular responses in HT29/c1 cells. (**A**) Western blot analysis of HER2 expression in WT, KO#1, and KO#2 HT29/c1 cells. (**B**) Immunofluorescence staining showing the absence of HER2 in KO clones. HER2 is shown in red and nuclei in blue (DAPI). Scale bar: 10 μm. (**C**) WT and HER2 KO cells were treated with rNT or rET (1:10 dilution in serum-free media) for 6 h. Cell lysates were analyzed by Western blot for E-cadherin, EGFR, and HER3. (**D**) IL-8 secretion was measured by ELISA after 24 h of rNT or rET treatment. Data are presented as the mean ± SEM. *** *p* < 0.001, ** *p* < 0.01. (**E**) WT and HER2 KO cells were treated with rNT or rET for 40 min, and ERK signaling was analyzed by Western blot. GAPDH was used as a loading control.

## Data Availability

The original contributions presented in this study are included in the article. Further inquiries can be directed to the corresponding author.

## Abbreviations

The following abbreviations are used in this manuscript:

ADAM10	A disintegrin and metalloproteinase domain-containing protein 10
*B. fragilis*	*Bacteroides fragilis*
BFT	*Bacteroides fragilis* toxin
BHIB	Brain heart infusion broth
CRISPR	Clustered regularly interspaced short palindromic repeats
Cas9	CRISPR-associated protein 9
ETBF	Enterotoxigenic *Bacteroides fragilis*
ERK	Extracellular signal-Regulated Kinase
HER2	Human epidermal growth factor receptor 2
MAPK	Mitogen-activated protein kinase
MMP-7	Matrix metalloproteinase-7
rNTBF	Recombinant non-enterotoxigenic *Bacteroides fragilis*
rETBF	Recombinant enterotoxigenic *Bacteroides fragilis*
SiRNA	Small interfering RNA
SFM	Serum-free medium
